# Supporting Remote Social Robot Design Collaboration with Online Canvases: Lessons Learned from Facilitators’ and Participants’ Experiences

**DOI:** 10.1007/s12369-023-00966-6

**Published:** 2023-01-30

**Authors:** Aino Ahtinen, Kirsikka Kaipainen, Salla Jarske, Kaisa Väänänen

**Affiliations:** Tampere University, Computing Sciences, Tampere, Finland

**Keywords:** Online collaboration, Collaborative design, Interaction, Teamwork, Human-centred design, Social robots, Facilitation

## Abstract

Social robot design projects typically involve multidisciplinary teamwork and collaboration, adopt a Human-Centred Design (HCD) approach, and deal with physical (tangible) objects, i.e., robots. HCD takes a human to the centre point of the design process. A typical activity in HCD are design workshops where a facilitator is needed to guide and moderate the task-related and interactional activities throughout the session. Facilitation is also usually needed in longer-term design projects or courses to guide participants through the different phases of design during several sessions. Recently, due to the COVID-19 pandemic, most design activities including social robot design were rapidly transferred to online mode. Designing for tangible objects is challenging in online settings because the interaction experience with a physical object is hard to demonstrate online. In this article, we report how we harnessed online canvases to support both short-term social robot design workshops and a long-term design course. Based on participants’ feedback and facilitators’ experiences, we report lessons learned from utilizing collaborative design canvases for creative social robot design projects that specifically focus on early stages and concept ideation. We propose practical guidelines for canvas-based online facilitation focusing on creative design workshops and projects. In addition, we discuss the lessons learned concerning social robot design activities taking place in online mode. To respond to the challenges of designing tangible robots in a fully online mode, we suggest a Hybrid Robotic Design Model (HRDM), where the participants work in contact with facilitators, other participants and robots at specific points, while the other phases are conducted online.

## Introduction

Social robots are considered and already used for various purposes in society, including education [1] and healthcare [2]. According to a commonly used definition, a social robot can be defined as “*an autonomous or semi-autonomous robot that interacts and communicates with humans by following the behavioral norms expected by the people with whom the robot is intended to interact*” [3]. However, a recent definition by Fox and Gambino [4] emphasizes a more realistic conception regarding social robots’ human-like communication skills and covers both physical and virtual entities: “*… technologies that can take physical or digital form, resemble people in form or behavior to some degree, and are designed to communicate with people.*” In this article, we focus on the design of physical social robots, because physical embodiment plays a strong role in acceptance, interaction and feasibility of social robots especially in social tasks [5].

This article reports lessons learnt when shifting from face-to-face human-centred social robotics design projects and workshops to remote mode, focusing on facilitation experiences and participant feedback. In the early 2020, the COVID-19 pandemic forced many design projects and workshops to be shifted into an online mode, setting new and unforeseen challenges in how to remotely facilitate the design process and collaboration. A remote setup can be especially challenging when the object of the design relates to tangible objects, such as physically embodied robots, as design of physical objects requires understanding of embodiment and interaction with the object. Human–robot interaction is more versatile than interaction with e.g., an app or a web service, as interaction with a robot typically utilizes different modalities such as voice, touch, and vision. Physical embodiment of robots enables, for example, movement in space, taking eye contact and physically touching an object, which are unique design aspects for physical objects.

Facilitation of creative design projects and workshops is a typical activity in the fields of interaction design, service design and human-centred design. Typically, these kinds of facilitated design-oriented workshops or longer-term projects rely on multi-disciplinary teamwork with pre-defined goals of designing an interactive application, service, or other type of a digital product. Facilitating and running creative design projects can be demanding for the facilitator, who needs to lead not only the design part of the project, the methods, the flow, and the tools in use (task facilitation), but also take care of team collaboration and overall atmosphere (interaction facilitation) [6]. Visual online collaboration tools in design can enable fluent interaction and flexibility in a hybrid setting, but their use in purely online mode needs further investigation [7]. Previous research has highlighted the need for easy collaboration and communication of the participants and the facilitators, and the value of the cloud-based design-specific software for visual communication [7–9]. However, as stated by Fleischmann [7]: “*More research on the use of cloud-based visual online collaboration tools needs to be conducted to validate its usefulness in an online delivery mode*”*.*

In this article, we describe and reflect on what we have learned from utilizing *canvas-based online facilitation* in several short-term robotic design workshops and a longer-term design course related to social robotics. Overall, these design activities concentrated on the early stages of design including ideation and conceptual design. This article addresses the following research questions:

1. How did the online canvas tool support teamwork in remote social robot design projects?
2. What are the practical guidelines for canvas-based facilitation of collaborative online design activity?

We focus on both participation and facilitation experiences, and based on them, *formulate practical guidelines for planning and conducting online design workshops and projects*. In addition, we suggest *a Hybrid Robotics Design Model (HRDM)*, in which participants work in contact with other people (facilitators and participants) and robots during specific phases, while other phases are conducted online. By presenting this model, we aim at overcoming the challenge related to designing tangible objects purely online. This is relevant, because it can be assumed that the pandemic will have a strong impact on what the “new normal” will be. It can be assumed that our working habits are changing at least partly, and even creative design projects that deal with tangible objects will be carried out if not fully remotely, then at least in hybrid mode including remote and face-to-face work phases [10]. The change in work habits relates also to accessibility, inclusiveness and sustainability of designers’ work, as online work can enable wider access to participation for different stakeholders in co-design work. Remote work also obviously supports environmental sustainability as travel emissions are greatly reduced.

## Related Work

In this section, we present related work in four sub-sections. First, we explain what Human-Centred Design (HCD) is and how this approach can be used in social robotics design projects. Second, we describe what is meant by facilitation of design activities, and what skills and activities are needed from the facilitator. Third, we present what kinds of tools are typically used in design facilitation. Fourth, we review what is already known about online facilitation.

### Human-Centred Design of Social Robots

In the design of social robots or their applications, Human-Centred Design (HCD) process can be applied. According to ISO 9241-210 standard [11], Human-Centred Design focuses on users, their needs and requirements, and aims to enhance effectiveness and efficiency and improve human well-being, satisfaction, accessibility and sustainability [11]. A key component in HCD is to engage people throughout the design process [12] by utilizing several phases in iterative manner: planning, understanding the context, specifying the user requirements, producing the design solutions, and evaluation of the solutions [11, 13].

Concerning the design of robots, Axelsson and colleagues [14] present a user-centred participatory design process for social robots. Their process consists of three phases: (1)* Defining the problem*, (2)* Creating guidelines,* and (3)* Defining the solution.* The process is supposed to be iterative, and user feedback plays an important role in the design work. Another process is defined by Tonkin and colleagues [15] who suggest a model of user experience design for Human–Robot Interaction (HRI) with eleven phases, starting from *“Challenge defining”* the last phase being “*Persevere, Pivot or Perish”*, and after that continuing with further iterations. Regardless of the model and the number of its phases, both models first aim to explore the user needs and expectations, define the guidelines or implications to lead the design work, design the solutions to respond to the user needs, and evaluate the solution with target users. The process is iterative so that the design is evaluated with the target users and refined based on their feedback, ideally with several iterative rounds.

Human-centred design has been utilized in several social robotics projects. For example, Björling and Rose [12] used HCD process in a co-design project with teens. There, they designed a social robot to support the mental health of teens. In their article, they present creative and age-appropriate methods to gather contextually valid data from teens, for example ideation by drawing, sketching, storyboarding and mock-up prototyping. They also present participatory research principles appropriate for designing new technologies with vulnerable populations. They emphasize that *“working with vulnerable population requires trust and authenticity in order to maintain community relationships”* (p.11). They also point out that the selection of the methods must include meaningful and ethical engagement for participants. They also recommend that research teams could benefit from the development of their own set of participatory research principles that maintain consistency within their research and provide ethical and transparent rationale for the research.

### Facilitation

Facilitation means “*interventions performed by a facilitator in a collaborative process that guide groups to achieve their common goals*” [16]. The role of a facilitator is challenging and requires several different skills and abilities. Facilitation may lead to high cognitive load of the facilitator during the session [16]. Among others, the facilitator needs to keep the focus of the working team on the task, create a positive dialogue environment, encourage participation, recognize different individuals and approach them individually [17]. Additionally, facilitation techniques need to be adjusted situationally during the session, as techniques that work with one group may not work with another kind of group.

Stewart [18] presents an extensive model of competencies required from a skilled facilitator. *Interpersonal competency* (communication skills) includes aspects such as verbal, non-verbal and written communication, listening, empathy, summarizing and paraphrasing. Interpersonal competency also includes further skills such as sensing/intuition, cultural awareness, flexibility, managing conflict, motivating and encouraging, as well as recognition of the achievement. *Management process competency* includes aspects like planning and organizing, time management, management of the tools and environment, management of the process interventions and feedback. *Personal characteristics* refer to the facilitator’s personal features such as adaptability, objectivity, self-confidence, sense of humour and management of personal energy. *Knowledge competency* refers to understanding the organizational context and the theory of group facilitation.

Group facilitation can be utilized in several areas, such as leadership [19]. Szumal [6] recognizes two different types of facilitation in leadership facilitation: interaction facilitation and task facilitation. *Interaction facilitation* is a skill of supervisors to utilize people-oriented skills and qualities to encourage supportive, cooperative interactions among their subordinates, thus supporting effective work performance in teams [6]. Thus, interaction facilitation means the facilitation of social dynamics, communication, and relationships. Sonnenburg [20] states that communication is the driving force for collaborative creativity. Sharing and criticizing are important aspects on encouraging creativity [16]. On the other hand, trust is also very important factor on effective teamwork [21]. In a qualitative study about the co-learning experiences conducted by Tseng and colleagues [21] participants reported that for example familiarity with the team members, commitment towards the high quality of work, and team cohesion were important factors for building trust with team members. Their study showed that teamwork trust correlated significantly with two of the important factors for building trust indicated by team members: familiarity with members (r = 0.74) and team cohesion (r = 0.79). *Task facilitation*, on the other hand, is the supervisor’s ability to facilitate the work performance of their subordinates by assisting them in problem-solving and in the implementation of procedural improvements [6]. Thus, task facilitation refers to the management of the goal, task and process itself. Both aspects are important in successful facilitation.

One area where facilitation is applied is called *design facilitation* [22, 23]. Design facilitation takes place in human-centred design projects, where human beings, i.e., the potential users of the upcoming products, are involved in the design process, and the designs are generated with the users. These co-design processes need to be facilitated, and this activity is called design facilitation. Design facilitation typically takes place in participatory design workshops or brainstorming sessions, where the designers, possible target users of the product and other stakeholders join a session that has specific goals and targets [24, 25]. Design facilitation is one of the seven emerging roles for designers working for the social good [26].

*Communication* and *presence* are essential aspects in creative design projects. The importance of the successful communication and collaboration has already been discussed in the previous section, and one of the main tasks of the facilitator is to support that as part of facilitation activity [6]. True collaboration needs active participation and dialogue within the participants of the group [27]. *Presence* is a psychological concept, which can be defined in many different ways [28]. In our work, we adopt the most suitable definition of presence from the perspective of being together in a shared virtual space. Based on this definition, presence is *“the degree to which participants of a telemeeting get the impression of sharing space with interlocutors who are at a remote physical site”* [29]. The feeling of being present is an essential part of teamwork and communication, which act as cornerstones of the success of these creative design projects. With successful facilitation we aim to increase the level of perceived presence of the participants by aiming for good facilitation of the atmosphere and social dynamics. The presence of the team members and facilitator play a strong role in the co-design projects. Robotics design projects include also another dimension of presence—the presence of the robots, which are tangible technologies. The physical presence of robots is missing from online robotic design projects, which create another challenging aspect for the design project.

### Tools for Design Facilitation

Different kinds of tangible hands-on tools are often used to support the design facilitation. Aguirre et al. [23] present three groups of tools that are typically used in design facilitation. First group of tools, *readymade tools*, involve typically off-the-shelf products such as sticky notes, big paper rolls, permanent markers, whiteboards, and flipcharts. These are general tools that are used for planning and analysing the design events, as well as spontaneously during the events. *Templated tools* have a pre-defined format, and they support in structuring the design event and the produced output in an effective way. Business model canvases, service blueprints, and SWOT analysis templates are examples of templated tools. Napier and Wada [22] present a canvas-based framework to support design facilitation, and their canvas can be used for planning and preparing for design facilitation tasks. The canvas includes six sections: People, Time, Environment, Methods, Tools (to make) and Supplies (to take). Another example of templated tools is a Triple Layered Business Model Canvas for exploring sustainability-oriented business model innovations [30]. The third group of tools include *contextually designed facilitation tools*, which are tailor-made tools for specific events [23]. These can include for example specific icebreakers, journey maps, and design cards for specific purposes. One example of contextually designed facilitation tools is a design toolkit developed for travellers’ experience design including passenger personas, context cards and passenger journey map [31]. Based on the amount of the tools for design facilitation, we can conclude that different kinds of facilitation and inspirational tools play a strong role in the successful facilitation of the creative human-centred design projects.

As already mentioned, different types of design canvases and templates are often used to support design facilitation and design projects. Our research utilizes the social robotics design canvases created by Axelsson et al. [14] as part of their social robotics participatory design framework. These canvases have been designed to support designers and researchers in the social robot design process, and they can be used for facilitating the discussion of the team members when they are designing a social robot. The canvases include, e.g., The Problem Space Canvas, where the “background” and the user needs for the design project are defined, and Minimum Viable Product Canvas, where the robotic solution for the identified needs is defined. In face-to-face design projects taking place in shared physical world, these canvases are printed out on large sheets of paper, and the design team works together with the tasks explained on the canvases. The design process is typically iterative, taking several re-design rounds based on the user feedback collected on each round. Based on the evaluation by Axelsson et al. [14], these canvases have several benefits for social robotics design projects. First, the participants considered the canvases providing structure, clarity and clear process to the design project. Second, the canvases encouraged different stakeholders and participants to share their different viewpoints when progressing towards the shared goal. Third, the canvases were perceived to provide an educational and enjoyable design experience for the design teams. In addition, according to the evaluation, the participants emphasized the role of the facilitator to support the design project. Axelsson et al. [14] mention that the canvases are best used with the help of a facilitator, who is familiar with the tool. The facilitator has an important role in going through the parts of the canvases, and explaining things when needed, as well as making sure that the team work together, and uses the canvases as intended. However, Axelsson et al. [14] emphasize also that the users of the canvases are free to adopt the canvases based on their needs. Some parts of the canvas may not be relevant for all design projects, and those parts can be skipped or adapted for the team’s purposes. The facilitator has a role also in the adaptation of the canvases. They mention that: “*We encourage future researchers to leverage the knowledge provided by the tool and the two example projects and make modifications to the canvas tools and the design processes as they see fit*” [14].

### Online Learning and Facilitation

During the COVID-19 pandemic, most of the design courses and workshops have been organized in online settings. Already before the pandemic, online learning was a well-established field with plenty of published academic research. One well-known and utilized model of online learning is The Five Stage Model [32–34]. In the model, the basis for learning is set in the first phase, *Access and motivation*, that emphasizes the importance of providing support for setting up and accessing technical systems as well as welcoming and encouraging learners. In the second phase, *Online team building*, effort is made to familiarize participants with each other and to bridge the gap between their cultural and social differences. After these two highly important initial phases, comes the third phase, *Information exchange*, in which the teacher acts as a tutor and supports the learners with learning materials. In the fourth phase, *Knowledge construction*, the teachers act in the role of facilitators towards the creation of knowledge, insights, and conclusions. The last phase, *Development*, consists of applying and integrating the learnings to the learners’ own contexts and tasks. The model puts a strong emphasis on the early stages of the learning process and highlights the importance of social and technical support when initiating remote learning.

Online workshop facilitation requires adjusting the methods and tools from those used in face-to-face workshops. As Park and Lim [35] describe, meaningful and successful online learning requires the establishment of a supportive learning environment, which is provided for participants via technology. Park and Lim [35] emphasize the meaning of designing an online learning environment that supports learners’ positive feelings (belonginess, empathy) and decreases negative feelings, such as feeling of begin isolated, frustration, boredom and anxiety. They propose a set of design principles to support their goal. For example, the principles of *positivity* and *playfulness* refer to the positive imagination and abilities to play individually and collaboratively while learning. Principle of *humanity* means delivering a sympathetic instructor formulation with feedback taking into account the human side. *Self-disclosure* refers to the possibilities to feel free about delivering a personal opinion, story or challenges. *Personalization* relates to the learners’ freedom to have flexibility in learning inside the environment, and *affinity* means designing an attractive environment which visually favorable impression. Principle of *safety* means that there should be enough technical support available for the students in order to manage the usage of different tools. It can be assumed that these factors play even more important role for students and participants in creative and visual subjects, such as design. Amro [9] studied interior design students’ experiences during COVID lockdown and reported the importance of empathy and understanding between design education instructors and students. In addition, she stated that technological tools such as ConceptBoard, Canva and YouTube can well support the creativity of the students in online settings.

As already described, interaction and positive emotions play a strong role in learning and those aspects would need to be considered when using different online learning tools. Fleischmann [7] reports positive experiences of design students using a visual online collaboration tool ConceptBoard in blended learning in a service design course context. Their aim was to augment the blended learning environment to broaden students’ learning opportunities by using a visual online collaboration tool to support the iterative creative design process of students working outside the classroom hours in an online team situation. Based on their findings, most students were engaged by the tool’s visual approach to collaboration and the ability to post and receive comments in real time. Some students would have needed more technical support in the usage of the tool. Students valued the project management capability of the tool; it made it possible to visually track the project progress. Students also perceived that the visual online collaboration tool can instill confidence in teamwork—they felt connected to the project and their fellow team members by using the tool. Fleischmann [7] concluded that these kind of visual collaboration tools can be valuable for design pedagogy. However, they stated that more research on the use of these tools would be needed to validate their usefulness in an online delivery mode. Furthermore, Fleischmann [10, 36] discusses the shift of the contemporary design education from face-to-face mode to blended mode, thus providing more flexible learning opportunities on the design field, and utilizing the meaningful aspects of online and hands-on modes.

Galabo et al. [8] present a study of a redesigning a physical workshop into a virtual one. They illustrate the application of a set of principles for designing and running co-design events online. They provide a practical set of guidelines for designing and running co-design workshops online. In their guidelines, they emphasize the good planning of the workshop and its activities. The activities on the online workshop need to be shorter than in the real-world workshop, as the attention span in online environment is typically shorter. They suggest activities that require participants to move a bit to be included as part of the workshops. Icebreakers would be beneficial in the beginning of the session to get people to talk. The importance of the technical facilitator is emphasized as well as very active facilitation and support during the workshop. They designed online canvases to structure the actual workshop tasks by using Miro board (miro.com). These canvases included the order of the design tasks, the suggested time to be used for each task, and the instructions of the tasks. The canvases were used together with Microsoft Teams.

### Summary of the Related Work

Based on our literature review and concerning our research focus, we can learn that human-centred design (HCD) is an applicable approach for social robotics design projects. An important part of HCD includes different kinds of group-based co-design workshops and other events, which needs a facilitator. The facilitator needs several skills, for example interaction facilitation and task facilitation competences. The experiences of successful communication and presence play a strong role in a pleasurable facilitation and participation experience. For online facilitation, there exist different technical tools for collaboration. However, the design projects which focus on designing tangible objects, such as physical robots, have additional challenges in their transition into online mode. It is challenging to demonstrate the physical appearance, embodiment, and interaction of the physical robot in online settings. Thus, more research is needed about the experiences and practices of how to conduct online design projects and workshops which focus on physical robots.

## Methodology

The research data were collected in two different types of projects (see Table 1): (1) short-term social robot design projects including 1–2 design workshops for high school and university students, and (2) long-term design project that was conducted as a university level social robotics design course lasting for 7 weeks. The short-term projects were conducted in in the local language of the participants (Finnish) and the long-term project ran in English, as the participants were international. The data were collected with a mixed-method approach by utilizing recorded debrief discussions and individual written reflections by the facilitators, background and feedback questionnaires and written reflections from the participants, and the canvases created with the online visual collaboration platform (Mural). The qualitative data were analysed with the means of content analysis [37], and the quantitative data with descriptive statistical methods.

**Table 1:** Characteristics of the design projects and study participants

Project	Project type	Participant profile	Time of organization	Duration	Number of participants	Age range	Data collection methods
University workshop	Short-term	University students (design)	May 2020	2.5 h	7	21–41 (mean 28.7, SD 7.9)	Questionnaires, canvases
University course project	Long-term	University students (user experience and technology)	October–December 2020	7 weeks, weekly 3-h sessions	19	N/A^a^	Students’ reflections, teacher’s reflection, questionnaire, course statistics
High school workshop 1	Short-term	High school students	December 2020	3 h (2 sessions, 1.5 h each)	12	17–20 (mean 17.4, SD 0.9)	Questionnaires, canvases, facilitators’ reflections
High school workshop 2	Short-term	High school students	December 2020 to January 2021	3 h (2 sessions, 1.5 h each)	6	17–20 (mean 17.7, SD 1.2)	Questionnaires, canvases, facilitators’ reflections

^a^Demographic data were not collected from the participants due to privacy requirements. Based on the students’ stage in university studies, we assume that most of them were between 20 and 30 years old

### Short-Term Projects: Design Workshops

#### Study Context and Participants

We conducted three online design workshops: one in May 2020 and two in December 2020. Participants in the first workshop were design students in a Finnish university (n = 7), and the participants in the second and third workshops were Finnish high school students (total n = 18). The workshops were part of a series of co-design workshops with the purpose for ideating social robot concepts in the context of youth civic participation. The university workshop was a one-time, 2.5-h event, whereas the high school workshops were both spread into two 1.5-h sessions held on two separate days to fit the participants’ school schedules. The university workshop had three facilitators (first three authors of this paper) and the high school workshops had four facilitators (the first three authors of this paper and another researcher). One of the facilitators (the second author of this paper) had the main responsibility for technical support in the workshops.

The structure of all workshops included five parts: (1) welcome and introductions, (2) overview of the topic and formation of groups, (3) ideation of the robot’s use cases, (4) ideation of the robot’s appearance and activities, and (5) feedback and closing. Parts 3 and 4 included short wrap-ups of design outcomes. Participants filled in a background questionnaire before the workshop, and a feedback questionnaire at the end of the workshop.

#### Workshop Canvases

**Fig. 1:**
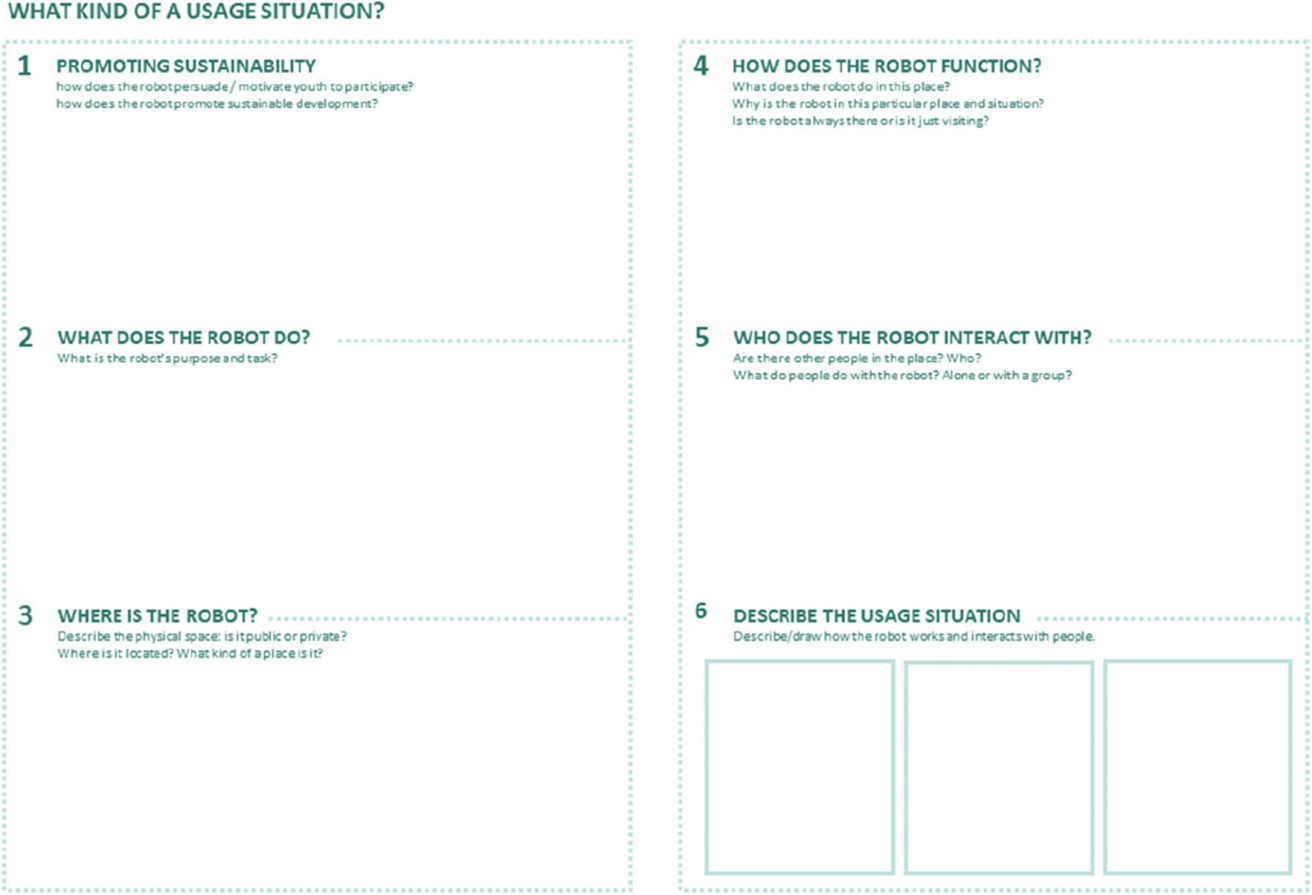
The first section of the two-part paper canvas used in face-to-face workshops before the COVID-19 pandemic. The language of the original canvas was Finnish

The workshops were based on a protocol used in earlier face-to-face workshops before the COVID-19 pandemic. In the original protocol, the workshops took place in a shared physical space and the participants were divided into groups of three to five people. To support the design process, we developed a design canvas inspired by the social robot co-design canvases by Axelsson et al. [14]. The canvas consisted of two parts: the usage situation (see Fig. 1) and the robot’s characteristics.

**Fig. 2:**
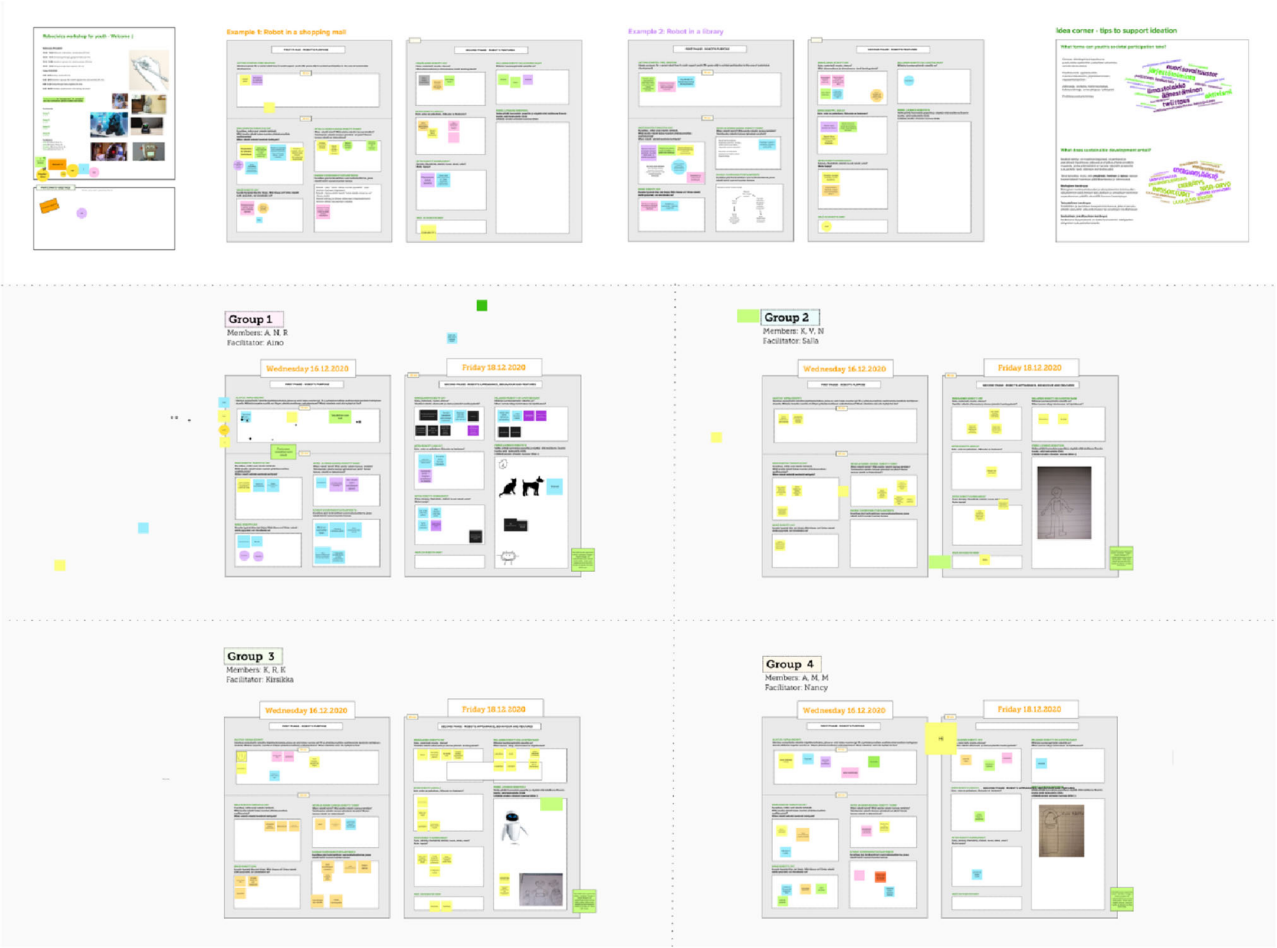
Example of a canvas used in an online co-design workshop. The contents of the canvas have been partially translated from Finnish into English and participant data have been anonymized

As we converted the workshops into online format, we wanted to replicate the original protocol as much as possible. For this, we prepared the design canvases online using the Mural platform (https://mural.co/). To mimic the experience of being in the same space with other groups, we laid out multiple design canvases on the same single base canvas, allowing each group member to navigate in the same virtual space (see Fig. 2). For communication, we invited participants to a Zoom meeting for shared discussions and used the breakout room feature to separate participants into smaller groups during group work. During group work, participants were assigned a task of creating a sketch of their design, and participants were given option to use the drawing tools provided by the platform or sketching their design on paper and uploading that image to the shared canvas.

#### Data Collection

Data were collected from workshop participants with online questionnaires. Participants were asked to complete a background questionnaire before the workshop, and a feedback questionnaire about participation experience at the end of the workshop. The feedback questionnaire included a question about workshop experience with ten quantitative statements on a 7-point disagree-agree scale, and qualitative questions about what participants learned in the workshop, what were the most important things in the designed robot, possible advantages of social robots, and open feedback about the workshop.

Data concerning facilitators’ experiences were collected from recorded debrief discussions and individual written reflections by the facilitators. The data collection method resembled autoethnographical data collection [38] in that facilitators reflected their experiences, private thoughts and actions.

### Long-Term Project: University Course Design Project

#### Study Context and Participants

The long-term design project took place at Tampere University’s social robotics course in autumn 2020 during the pandemics. The main goal of the course was to get familiar with different kinds of social robots, get to know the state-of-the-art related research, and conduct a social robotics related design project as teamwork. 19 university students participated in the course. The participating students were from different disciplines, i.e., human-technology interaction and robotics. The course lasted for seven weeks and was running mainly online. The students, who could enter campus were given possibilities to experience the robots in action and program them in small groups. Five students came to the campus to see the robots in action in the beginning of the course, and six students came to campus to program the robot. The project topics were selected by the teams. Several topics were available, and in addition to that, the students could suggest own topics. The teams of 2–4 members were formed in the first session of the course. In total seven teams were formed based on the interest towards different topics.

The online tools that were used in the project were: (1) Zoom conferencing software for the meetings including common room and breakout room discussions, (2) Mural canvas for the project tasks and documentation, (3) StoryboardThat concepting tool for visualizing the concept ideas and (4) tools selected by the participants for the internal communication and information sharing (e.g. Telegram, Google docs, OneDrive).

#### Project Canvases

**Fig. 3:**
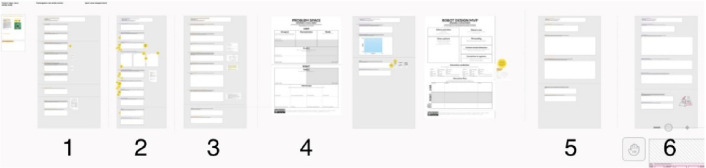
Social robotics design course phases shown in separate canvas slots from the topic selection and team formation, to the reporting and presenting of the project outcome. Phase 4 canvases”Problem space” and”Robot design MVP” by Axelsson et al. [14]

The project was facilitated by the course teacher (the first author of this paper) in weekly 3-h small group exercise sessions. In these sessions, we had a common session first for introducing the topics and tasks, and then the groups were divided into breakout rooms to work with the tasks. The students also worked in groups outside of the dedicated course hours. The project proceeded throughout the following human-centred design stages on a weekly basis: (1) topic selection, team formation, warm-up, project planning and state-of-the-art search, (2) planning and conducting a user needs study (online study) around the selected topic, (3) analyzing the data and formulating design implications, 4) concept brainstorming and design, (5) concept implementation (either by programming the robot at campus or designing a visual scenario), (6) planning and conducting an evaluation study, and (7) reporting and presenting. Thus, every week there was a specific learning theme, and the related activities were supposed to be conducted during that week. We created a digital canvas to support all the project’s stages. On the project canvas, each week had an own sub-canvas, which included the tasks of that specific week. The tasks were given on the canvas in a way that the larger task of the week was split into smaller activities. A suggested length for each task was provided on canvas. The idea was that the students were documenting their work on the canvas by using sticky notes. The facilitator followed the groups’ work on the canvases, gave feedback, questions and comments on the canvases as sticky notes, and entered the breakout room only on a needs basis, for example if she thought the team was stuck on something, or if the group asked for help. All groups were given their own canvas that was accessible only by the group members and the facilitator. Most canvases were created purely for the course purposes. Figure 3 shows an overview of the whole canvas including separate slots for each week’s topic and tasks.

For example, on the first weekly exercise some time was invested (suggested length: 15 min) for the establishment of team spirit and ground rules. The groups were divided into breakout rooms and they were given tasks about introducing themselves as well as creating explicit ground rules for the group. The tasks were given on canvas (Fig. 3), and as during the whole course, students discussed in the breakout room by voice and having their camera switched on if they wanted. They added the main points about the discussions on the canvas.

As another example, the fourth stage of the project was about concept design and implementation. On this phase, the students were ideating the robotic concept based on the design implications that were formulated from the pre-study findings and related literature learnings. The ideation started with the initial brainstorming task with “yes, and…” technique [39]. The task on canvas asked the students to brainstorm their concept in a positive and accepting approach and post all ideas on canvas. In the following task, they organized the ideas based on their feasibility and expected impact for the user. Stage 4 also involved the Problem Space and Robot Design MVP canvases by Axelsson et al. [14].

#### Group Formation Canvas

A separate canvas for the group formation was made. The groups were formed, and the project topics selected partly as a pre-assignment, partly during the first session of the course. The first session was an introductory lecture including the basics of the human–robot interaction as well as a briefing to the course’s requirements and work habits.

**Fig. 4:**
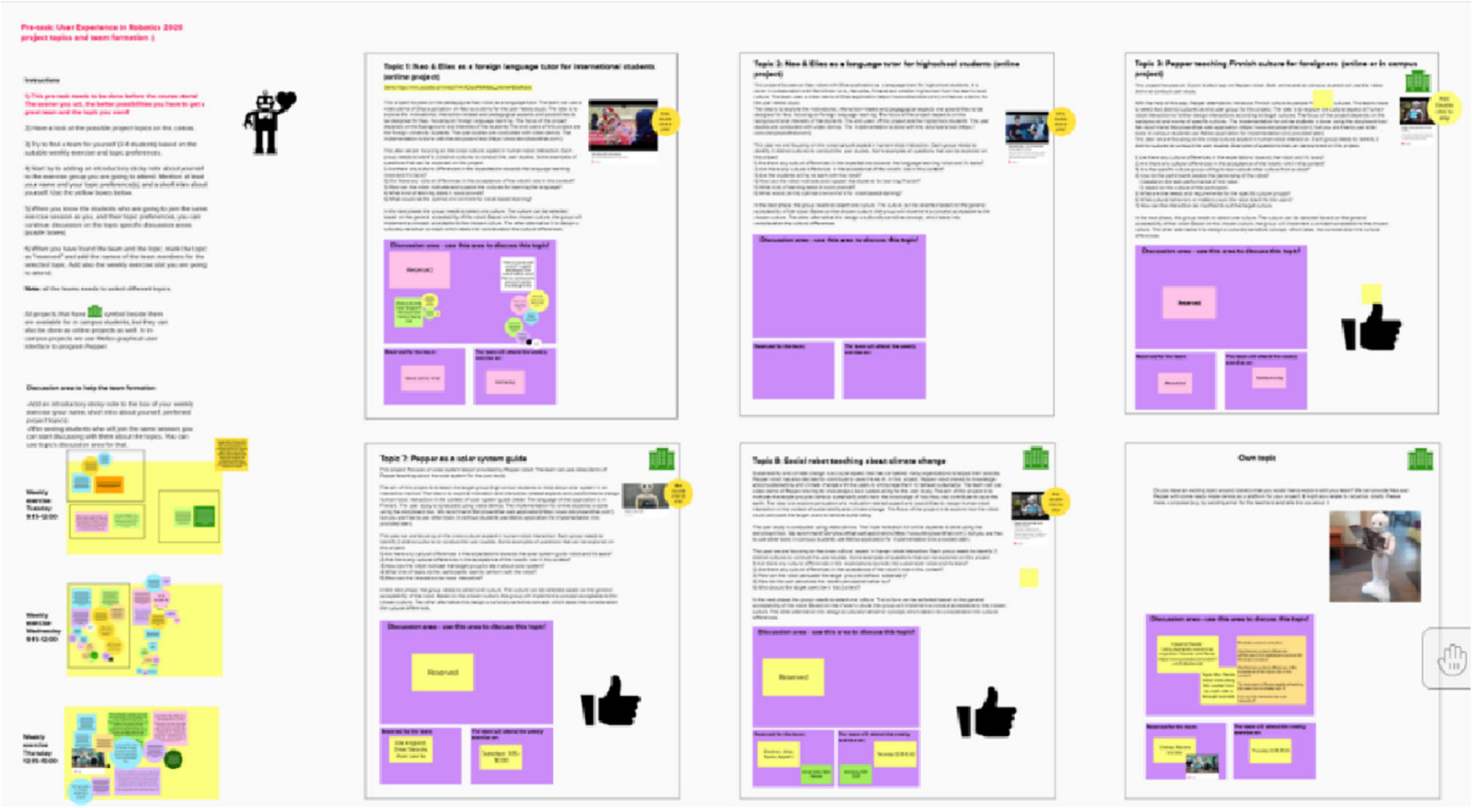
A canvas for the group formation including instructions for the task (left top corner), introductions made under the weekly exercise slots available (left bottom corner), and the slots introducing the project topics and the actual group formation (larger slots including purple boxes)

In practice, the group formation took place on the canvas, where all the available project topics were described with text and related video links. As a pre-assignment, the students got the link and instructions to the canvas, and they were instructed to view the topics and discuss them on the canvas in advance. Figure 4 shows part of the canvas that was used for topic selection and team formation. On the group formation canvas, the students first posted a short introduction about themselves and their project preference as a sticky note on one of the yellow boxes that represented the three alternative timeslots for the weekly exercise sessions. This was the initial step towards the group formation as the students could see who was going to join which session, read each other’s short introductions, as well as see everybody’s topic preferences. The boxes with purple squares represented available project topics. For each project topic, there was a textual description and a video link to demonstrate the topic. The students interested in a specific topic could discuss about the topic and group formation by adding sticky notes on the slot of the topic that they were interested in. After the group was formed through the sticky note discussion, the group added a note with the information that the group was ready, group member names, and their exercise session choice.

#### Storyboarding Implementation

**Fig. 5:**
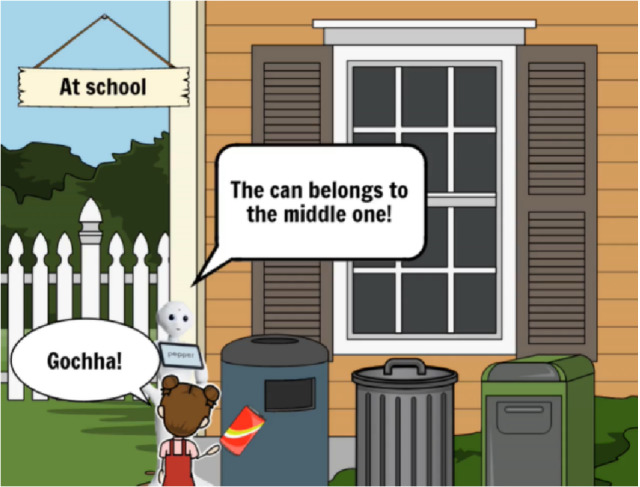
StoryboardThat online service was used for the robotic concept implementation

Due to the online study mode, we needed to make the concept implementation phase mostly in storyboarding technique, instead of implementations on the robotic platforms. Storyboarding suits best for initial phase visualization of the idea [40]. The StoryboardThat online service (https://www.storyboardthat.com/) was used for the concept implementation, and with that, the students visualized their concept idea by using a cartoon-like technique (Fig. 5).

#### Data Collection

The research data were collected from this group of participants by the means of text-based course reflections, which were written by the course teams and submitted as part of the project report. In the reflections, participants were asked to reflect in an open-ended manner on their learning experience, the online design process, their group’s collaboration, and the tools used. The reflections were typically 1–2 pages long. At the end of the course, the students filled in a feedback questionnaire (similar to the one collected in short-term design workshops) including both quantitative and qualitative questions. The course teacher also wrote a reflection of the course from the facilitator’s perspective. In addition, simple course statistics were used in the analysis, such as attendances to the sessions and course completion rate.

### Research Ethics and Data Security

For the high school workshops, ethical approval was acquired from the city officials. The study was planned in collaboration with a teacher from the participating high school, and students and their parents were informed of the study through the school. Participation was voluntary and informed consent was collected in the beginning of the background questionnaire before the workshops. For the university student workshop and the university course, informed consent was collected from the voluntary students. For the course students it was explained that participating or non-participating in the study would not affect the course performance or evaluation. Research data were collected only from those who gave their consent to participate in the study. All data were pseudonymized in the transcription phase, i.e., all direct identifiers were removed from the data, and the research data were stored in the secure drive provided by the university.

### Data Analysis

The study produced mainly qualitative data. The qualitative data were analysed with the means of content analysis [37]. The content analysis was conducted in inductive way, i.e., themes were identified from the data. Three researchers (the first three authors of this paper) took the responsibility of the content analysis in the way that there was one researcher named as a responsible researcher for each data set. The themes and findings were discussed and reflected among the researchers so that the final set of themes was agreed collectively. The quantitative data from the questionnaires were analysed with descriptive statistics. The data from the two high school workshops were pooled together for the analysis.

## Findings

In this chapter, we report the quantitative feedback from the participants of both short-term and long-term design projects, and then present the findings from the qualitative analysis of both participation and facilitation experiences in short-term and long-term projects.

### Summary of Participant Feedback

Table 2 summarizes the participants’ responses to the feedback questionnaires. On average, the experiences of the participants were on the positive side based on the feedback, and the facilitators appeared to have done their job well according to the participants. However, the experiences of the high school workshop participants were the least positive. This seemed to be mostly related to the subject matter of the workshop, since the statements that were rated lower concerned the interest in the topics, meaningfulness of participation in the workshop and willingness to participate in a similar workshop again. It is worth noting that although participation in the study was voluntary for all participants, the university students were more likely to participate due to their intrinsic interest in the topic than high school participants, since the high school participants were recruited through their teacher and the workshops were organized as a part of their coursework that was otherwise unrelated to robots or civic participation.

**Table 2:** Feedback survey responses. Results reported as mean (standard deviation). The scale of the statements was from 1 (strongly disagree) to 7 (strongly agree)

	Short-term		Long-term
Statement	University workshop (n = 7)	High school workshops (n = 15)	University course project (n = 16)
It felt meaningful to participate in the [workshop/project]	6.9 (0.4)	5.3 (1.0)	6.6 (0.6)
I liked the ways of working [in the project]	6.1 (1.2)	5.5 (0.8)	6.0 (1.0)
The atmosphere during the [workshop/project] was safe and open	6.9 (0.4)	6.2 (0.8)	6.8 (0.6)
My thoughts and opinions were heard	6.7 (0.5)	6.3 (0.7)	6.5 (0.5)
The structure of the [workshop/project] was clear	6.4 (1.1)	5.9 (1.1)	6.1 (0.9)
The facilitators of the [workshop/project] did their job well	6.9 (0.4)	6.5 (0.6)	6.6 (0.6)
I would participate again in a similar [workshop/project]	6.7 (0.5)	4.9 (1.5)	6.6 (0.8)
The [workshop/project] topics were interesting or important to me	5.9 (1.2)	4.0 (1.4)	6.5 (0.9)
I learned new things during the [workshop/project]	4.4 (2.0)	4.9 (1.6)	6.5 (0.8)
The tools used in the [workshop/project] were suitable for the purpose	6.9 (0.4)	5.7 (1.3)	(0.5)

### Findings from the Short-Term Social Robotics Design Workshops

In this section, we report the findings of the short-term projects, i.e., the university workshop and the two high school workshops, related to participation and facilitation experiences. The findings are reported under the broad themes of learning experiences and canvas-based collaborative design.

#### Learning Experiences

From the participant feedback, we recognized three categories of learning experiences: learning skills related to online collaborative design, gaining knowledge or interest to learn more about social robots, and activating one’s knowledge about societal participation (although the last category was present only in one university workshop participant’s response). One participant of the university workshop and four participants of high school workshops did not answer the question about learning experiences.

The design-related skills that some of the participants mentioned having learned included teamwork, using online tools (*“I learned to use Mural and how remote workshops work”*), presentation skills (*“I learned to speak better in front of an audience”*) and ideation (*“Coming up with new things for the future”*). These kinds of learnings were mentioned more often by the university workshop participants, whereas the high school workshop participants mentioned mostly robot-related learnings. The robot-related learnings were mostly stated in a concise and generic manner, e.g. *“I learned more about robotics”*, except for one university workshop participant’s response that connected social robots to a usage context: *“I learned the variety of possibilities how social robots could be used in social situations, and this aroused my interest in the subject more”*.

University groups were active and engaged in the project by themselves, whereas the high school groups needed to be guided more along the process, which meant that facilitators had to adjust their facilitation style to correspond with the needs of the group. Personal skills for responding to different situations during collaborative design were learned by facilitators. Regarding the workshop process, facilitators learned that more time is needed for group formation and warmups, specifically due to the slightly challenging topic of the workshop. Facilitators also felt that participants may need guidance during the concept generation phase to steer the discussion toward topic-relevant robot ideas.

#### Canvas-Based Collaborative Design

In the university workshop, the canvas structure was perceived by some participants as helpful in stimulating ideation and coming up with many ideas, e.g. *“The Mural canvas structure was prepared well, it was easy to answer questions and I didn’t get stuck in ideation”*. Most participants commented positively about the atmosphere of the workshop, using adjectives such as *“friendly”* and *“pleasant”*, and this appeared to make collaborative ideation easier: *“Facilitators were open and encouraging, and discussing in groups wasn’t difficult even for a student with anxiety”*. However, one participant considered the initiation and group formation phase insufficient and perceived that their group was ideating without a common vision: *“I’d have preferred to talk more about people’s thoughts about participation and sustainability. Now we started workshopping without a common thread.”* The first task for the groups was indeed about ideating purposes for a social robot, and the canvas structure did not include a discussion task about the subject matter. While the facilitators’ experiences of the university workshop were also positive, they recognized that the justification and selection of the idea that the groups would develop into a concept design were not based on a critical analysis of the ideas.

Several of the high school participants described the workshop overall as *“nice”*, but regarding canvas-based ideation, there was very little feedback. One participant commented that *“the best ideas come afterwards”*, possibly implicating that the time or structure for ideation was too limited or a break would have been beneficial. One participant also mentioned that while the workshop was good, the groups could be slightly bigger. This notion was also present in some of the facilitators’ reflections, as they experienced discomfort especially during the second part of the workshop when some of the group members had dropped out, but also in the ideation phase if the participants did not seem to be willing to speak out or place ideas on the canvas.

In online workshops, the practice of making oneself present turned out to be a foundational aspect. In the university workshop, the participants engaged in discussion and ideation after facilitators instructed them to start working as a group. In the high school workshops, the participants needed more structure and support to participate. From the facilitators’ perspective, uncertainty over the participants’ engagement during ideation phase in the online workshops added pressure on facilitation and was experienced as problematic. While the university workshop participants were engaged and active, several participants in the high school workshops did not speak or make their presence known to others by speaking or commenting. The participants remained silent after instructions were given, and facilitators experienced uncertainty. From the facilitators’ point of view, this prompted different strategies and practices to get participants communicating. Common to all strategies was the facilitators’ ability to keep trying regardless of personal or social discomfort.

The problem of communicative feedback in the high school workshops drew out the importance of active communication within the ideation groups. Facilitators experienced joy when participants would engage in discussion or even respond to a question. It became apparent in the facilitators’ reflection discussion, that any form of communicative feedback, or a sign of engagement in the ideation process (such as attaching a note on the canvas) was “celebrated”. Facilitators’ feeling of uncertainty reduced along each “unit of feedback” or “sign of engagement”. In their reflections, the facilitators acknowledged that communicating by speech may be more challenging and anxiety-evoking for younger students, and thus the facilitators tried to encourage also other forms of communication, especially post-it notes on the online canvas.

### Findings from the Long-Term Social Robotics Design Project

In this section we describe the findings about participation and facilitation in the long-term social robotics design project in the university course context. The section presents the findings in six categories: (1) the overall learning experience, (2) canvas-based group formation, (3) warm-up to teamwork, (4) documentation and project management, (5) canvas-based interaction, and (6) concept design. Authentic quotes are used to illustrate the findings. The quotes have been coded with the student group number, for example G1 refers to group 1.

#### Overall Online Learning Experiences

All groups in the robotics design course described their learning experience in the project as positive, as the following example quote from one student group illustrates: *“The project had successful and challenging aspects that made it an interesting experience for the team members. As a learning experience we considered that the project was successful.”* (G7). The students thought that they were able to learn about robots and robotics, as well as manage to successfully carry out the required activities, such as user interviews. Good learning outcome were typically raised in the reflections of the students: *“We didn’t have any experience with robots before and we feel like we learnt a lot…”* (G1). In addition to the robotics design process, they mentioned learning about the related design tools: *“In addition, we learned the design process and specific canvases that can be used to design the user experience with a robot.”* (G7). Some groups recognized that they had also learned about professional skills, such as team management and project management: *“The online project work was a new experience for all of us, but this experience also made us learn new things such as team management and project management.”* (G4). All 19 students who started the course completed it within the given time frame.

However, at least some of the students would have preferred face-to-face mode over the online mode, despite the positive feedback that they gave about the project: *“In an ideal situation we would have seen each other more often.”* (G1); *“We think that the process would have been much interesting to do face to face. But due to the COVID 19, this implementation of the coursework has been very manageable and easy to do.”* (G4); *“Nevertheless we succeeded in creating a coherent concept although it did take more time than expected when compared to face-to-face teamwork.”* (G5). According to these quotes, at least some students would have wanted to work together physically in the same space which would have made the project more interesting and smoother.

#### Canvas-Based Group Formation

**Fig. 6:**
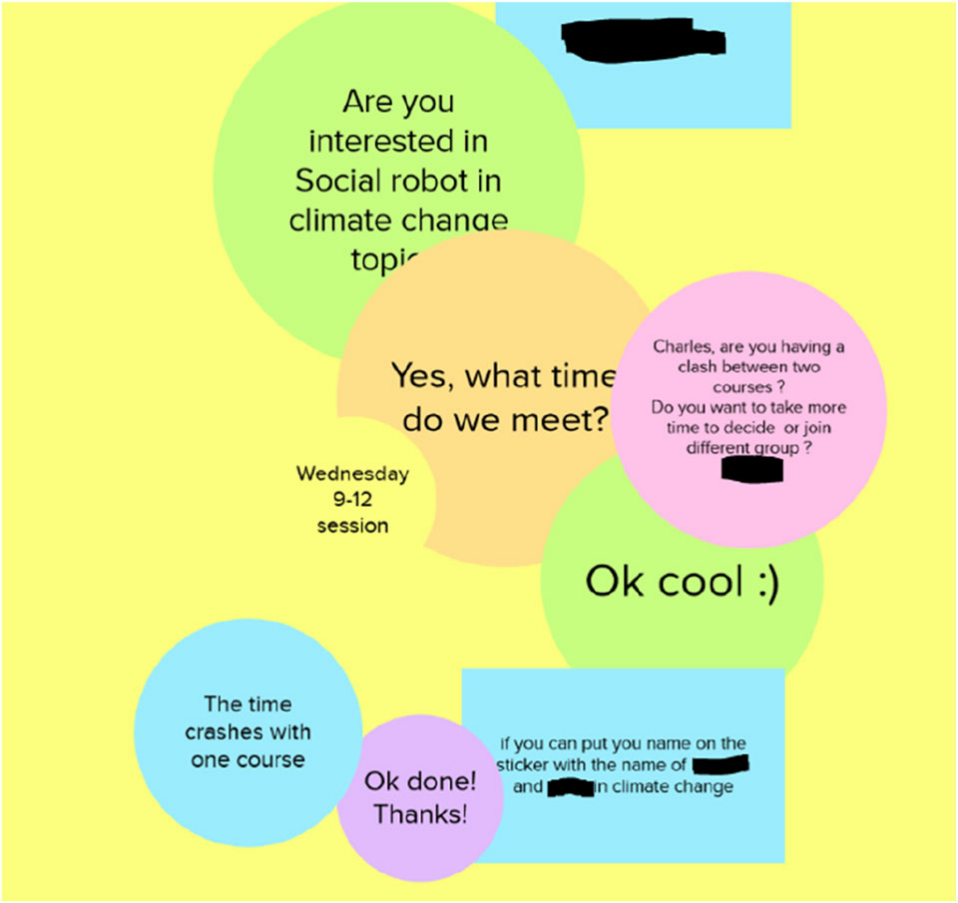
Students communicated on the group formation canvas about the group members, weekly exercise time slot, and the topic

The project groups were formulated on the canvas as a pre-task. By the first session of the course (the introductory lecture), most of the students had marked their names under their preferred topic on the group formation canvas, and some groups had already been formed independently before the first session. On the introductory lecture, some of the groups were still missing members, or some students did not have a group. We used half an hour in the session to finalize the group formation. If needed, the students willing to work in the same team, or the students who were still missing a group, were flexibly directed to a breakout room discussion to talk about group formation related matters with the potential group. All group formation issues were solved during the first session either by writing sticky notes on canvas or by having a discussion in a breakout room. For example, the students negotiated about the consistency of the groups, the suitable weekly exercise slot, and the preferred robotic topic as sticky notes (see Fig. 6). All the groups (in total seven groups) were formed by the end of the first session.

**Fig. 7:**
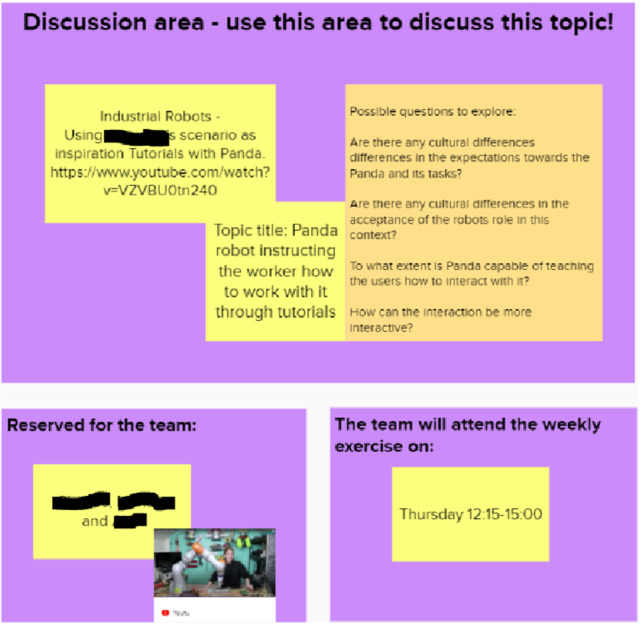
Part of the group-formation canvas showing the sticky notes posted by the group members

From the facilitator’s perspective, canvas-based group formation worked smoothly. The project topics were easy to describe and visualize on one big canvas, and the status of the group formation and selection of the topics was visible for all course members and staff. The students appeared to take responsibility to form the groups, and they seemed to be happy to be able to select the topic that they were interested in, but they were also ready to be flexible and discuss other possible topics. The facilitator’s role was to introduce the task, encourage group formation, and lead the students to discuss in breakout rooms if needed. All in all, the students formed the groups quite autonomously, and it seemed that seeing the most active students’ sticky notes on the canvas also encouraged others to add sticky notes on it. The canvas-based group formation seemed to be a beneficial task in sense that it set the students into an active mode, and at the same time they got familiar with the canvas-based activity already before the course started. Figure 7 shows an example of a sticky note-based discussion about one course topic, as also illustrating how group members and the selected exercise session were typically marked on the slot of the selected topic.

#### Warm-Up to Teamwork on Canvas

**Fig. 8:**
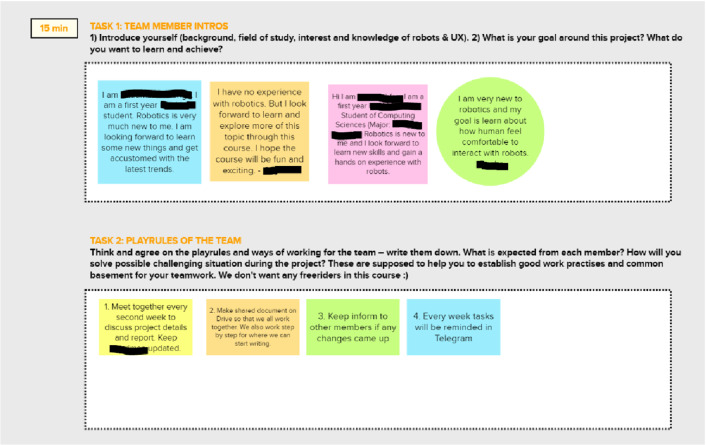
The warmup tasks with one group’s sticky notes

On the first exercise session, the group members got familiar with each other by making introductions and by creating ground rules for their work. These tasks were visible on the canvas. From the facilitator’s point of view, these warm-up tasks served very well as part of the grounding of the teamwork, and it was worthwhile to invest time in it before starting the actual work around the project topic. The canvas made the warm-up tasks explicit and clearly visible to the students. The comments posted on sticky notes kept the communication visible for the students as well as the facilitator. For the facilitator, it was easy to follow the progress in tasks, and give support or ask them to move on if they seemed to be stuck on some tasks. Even without explicit instruction, many groups formulated a colour-coding system for sticky notes so that a specific colour was reserved to each student. The specific style in making sticky notes can be seen as part of expressing one’s identity on the canvas. From these early project stages, the canvas started to look personal and unique for each group due to their own style of documentation and usage of the canvas. Figure 8 shows a screenshot of the warm-up tasks from one group’s canvas. The colour coding for each member is visible in the screenshot, as well as the group’s tendency to organize the sticky notes into a neat grid.

#### Canvas-Based Documentation and Project Management

The project activities were facilitated in weekly exercise sessions. It seems that the students were satisfied with the facilitated sessions, and they considered them supportive for their project: *“The exercise sessions proved to be helpful for all of us as they really helped us in planning and managing our project work.”* (G4). The students seemed to be satisfied with the facilitation methods that were used: *“The use of the facilitation methods was proved to be really helpful.”* (G4). As the following quote describes, these facilitated sessions seemed to form the backbone for the project management work: *“We used the course’s exercise sessions for ideation, analysis of the data, as well as distribution of the tasks*.” (G3).

From the facilitator’s perspective, these weekly sessions were central to the course – it was the forum where teams’ status was gone through, the week’s activities were presented, and where the main support from the course personnel was available. Students participated in these sessions actively. Based on the course statistics, the attendance to the exercises was almost 100%—three persons were absent from one session, one person was absent from two sessions. From the facilitator’s perspective, the atmosphere during these sessions were active, enthusiastic, and motivational, and the active work mode was visible through the canvas.

**Fig. 9:**
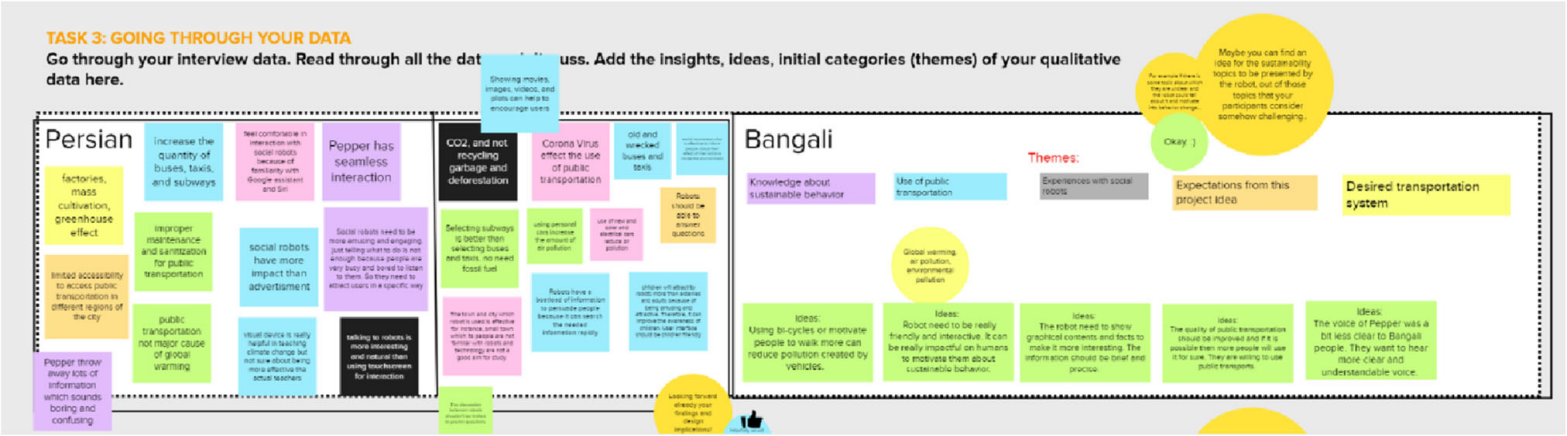
Active documentation of the project by using sticky notes on canvas

The canvas was used actively during each stage of the project. The Fig. 9 with sticky notes posted by the students illustrate how actively the canvas was typically used by the groups. They actively posted sticky notes for every task described in the canvas. The students documented the main points of discussion for each task on canvas. The sticky notes summarized the ideas, thoughts and discussion points concerning the tasks.

According to the students’ responses as well as facilitator’s reflection, the use of the canvases was one of the most successful aspects on the facilitation throughout the project. First, the students gave only positive feedback about them, which can be seen on the following comments: *“We absolutely loved to use Mural and Zoom at the same time to supplement each other really well.”* (G1);*”I would be willing to use Mural on other courses as well, and I think it is a great platform for making activities.”* (G2). One clear reason for why the canvas was liked so much, was its ability to support the management of the whole project and keep the information in one place: *“Especially mural was very informative as it helped us to maintain the project data and information till the end… The use of Mural made it easier for us to catch up the details if someone missed a session. It also helped us to find the important information all at one place.”* (G4) The students also stated that the canvas helped them to brainstorm and collect all the ideas that came out, as well as all related materials: *“Mural was a nice way to brainstorm and at the same time also note down the ideas and links we came about.”* (G5). Afterwards, it was easy to see how the project has been progressing, both for the students as well for the facilitator.

From the facilitator’s point of view, splitting the large project goal into clear weekly topics and smaller activities, as well as describing the project flow and tasks clearly on canvas, had a great benefit for the project work and management. The students were actively documenting their discussions on canvas, and they also actively posted external links, links for external documents etc. on the canvas. The canvas seemed to act as a comfortable home base, where all materials, thoughts and ideas were documented as a manageable manner.

During each week, the students could flexibly arrange their work as they wished, and plan, allocate and share their tasks according to the team’s schedules and available resources. While the weekly deadlines and clear tasks given on canvas set a clear framework and deadlines for the different phases of the project, it still allowed for flexibility, and thus demanding for time management and project management from the team members. This seemed to work: *“The project has proceeded smoothly. We have done it forward flexibly when we have had time to do it, and the workload has not felt overwhelming.”* (G3); *“We managed to do well in our teamwork considering the needs of every project member.”* (G4) *“Overall, our group worked well together, and we had no trouble keeping up with our planned schedule.”* (G7) It was mentioned that the canvas helped in organizing the project process: *“Mural assisted us in brainstorming and organizing a schedule for our future project procedure.”* (G6).

#### Canvas-Based Interaction

As already described, the students posted the main points out of their work on canvas as sticky notes, and the facilitator gave feedback and comments on the same way. The facilitator adopted a specific style for the comments, i.e., round shape and yellow colour, so that the facilitator’s sticky notes were easily recognizable. The style of the sticky note gave a possibility to express one’s canvas identity.

**Figures 10:**
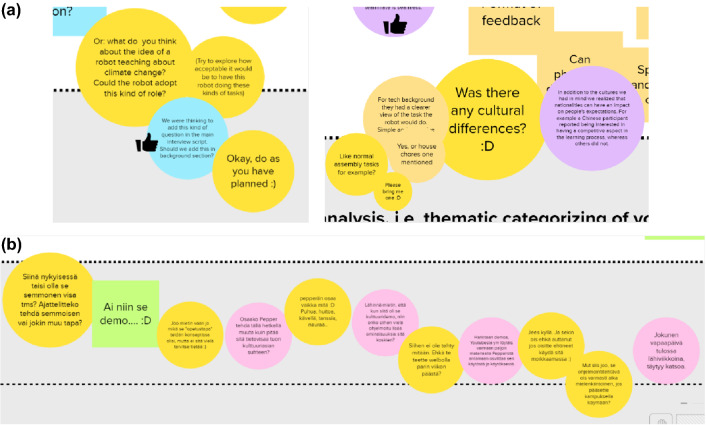
Canvas-based interaction between the students and the facilitator

From the facilitator’s perspective, the online canvas was a great way of “silent communication” with the students. Canvas and sticky notes provided a novel way to do the commenting in an unobtrusive way. Facilitators experienced the sudden entering to the breakout room as disturbing the work and stopping the flow of dialogue, while commenting on the canvas could be done more silently, still interactively. The students were willing to reply to the facilitator’s comments by using sticky notes, and typically, the students responded immediately to the facilitator’s comments (Fig. 10a, b). This kind of silent and interactive communication felt good from the facilitation point of view. It felt good to see the students working and adding sticky notes about their main points, and being able to comment on those in a novel and comfortable way. The facilitator also used symbols and emoticons on comments regularly, to add an emotional layer on the comments, and many times, the students did the same.

It was recognized by the facilitator, that the exchange of the colourful sticky notes, as well as viewing the students’ avatars moving on the canvas while they were working and documenting their work, felt dynamic and energetic. It was a good experience to perceive the presence of team members on the canvas and see activity taking place. Based on the facilitator’s reflection, it might have had also a positive effect on students’ motivation to work in the project, because of the inspirational nature of the canvas.

#### Canvas-Based Social Robot Concept Design

**Fig. 11:**
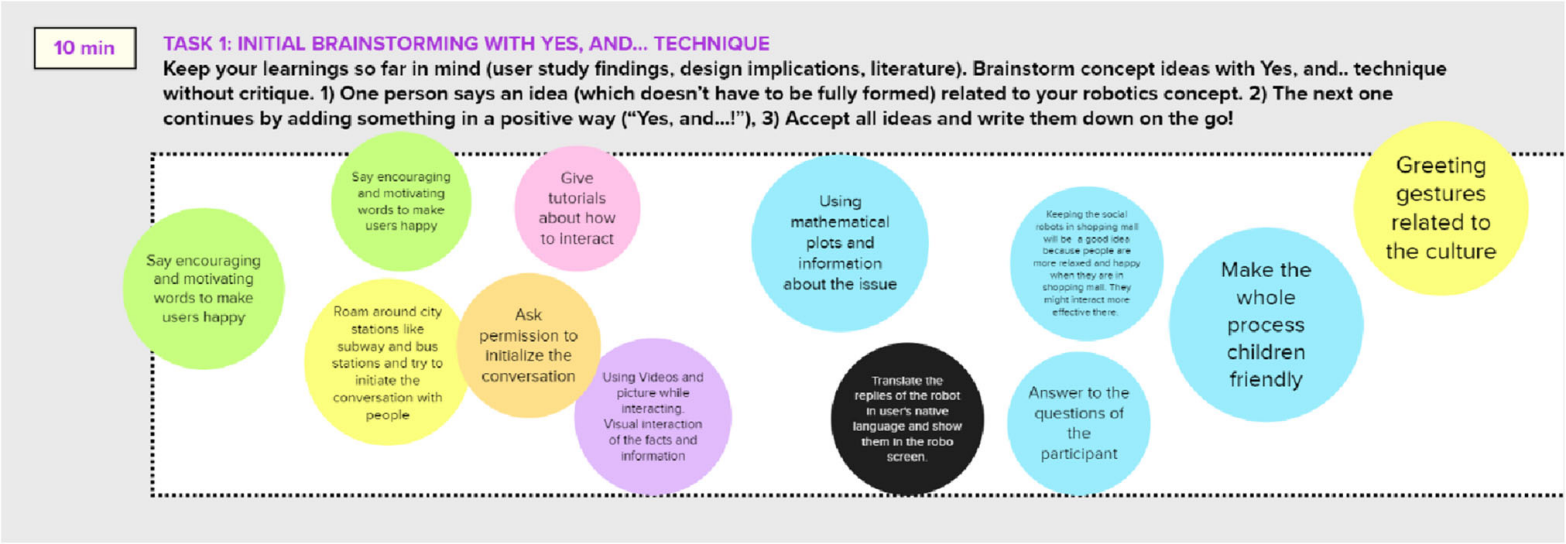
Initial brainstorming of the robotic concept with”Yes, and…” technique. The team members added all of their initial concept ideas to the canvas

**Fig. 12:**
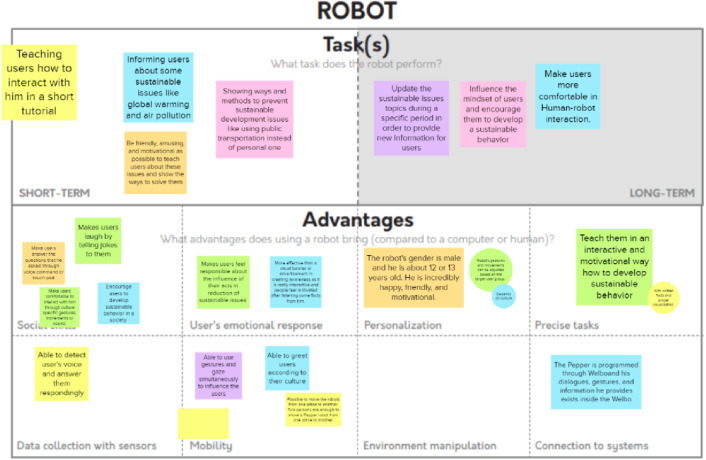
Part of the Problem Space canvas by Axelsson et al. [14]

The fourth stage of the project included robotic concept brainstorming and design tasks. In addition to our course-specific canvases, such as “Initial brainstorming with Yes, and… technique” (Fig. 11) we utilized “Problem space” as well as “Robot design MVP” canvases created by Axelsson et al. [14] (Fig. 12). The ideation and concept design canvases suited well for the concept design phase as they provided a tool and tasks for summarizing the user needs study and literature related findings, as well as a tool for the concept creation. The canvases worked well in online mode although they were originally developed for face-to-face design work. The students utilized the concept design canvases actively and flexibly for their purposes. They filled in the tasks with sticky notes in a very active manner but on the other hand they posted sticky notes only to the slots that were relevant to their project.

#### Robotic Prototyping

Not directly related to canvas-based collaboration, but more generally to working in online mode in robotics design, we present some related challenges focusing especially on the prototyping phase. We used storyboarding for the low-fidelity prototyping of the robot concepts with most of the teams, as they could not access the physical robots for implementation. Although the storyboards generated by the students were creative and visualized the ideas very well, this phase proved to be the most challenging part of the course. Storyboarding did not properly demonstrate the interaction between a human and a physical robot. Some students could access the robots at the campus and were able to make a proper implementation on the robot, and their comments reveal the importance of working with the physical robot: *“Programming the robot was maybe the most interesting phase of the project, in addition to the interviews. Although we had very limited time for this and we could only implement dialogues, it was interesting to work with the robot itself.”* (G3).

#### User Evaluation Phase

The same limitation of the online mode continued in the evaluation phase. The evaluation was conducted by showing the storyboard or a video about the concept implementation to the study participants, and by conducting an interview to get feedback. Some students perceived this as a limitation: *“Evaluating the prototype online was not as working [compared to the initial pre-study]. Especially because our evaluation did not involve any interaction [with the robot], but it was just a video.. when there is no interaction, the user is not able to experience the interaction, and the evaluator is not able to observe the user’s reactions during the interaction. Observation may bring in issues that are not revealed in the interview.”* (G3); *“Interaction is for sure very essential part of the use of a social robot.”* (G3); *“Some challenges included the current COVID-19 pandemic situation which prevents from evaluating the concept in person.”* (G7).

One group provided a good development idea for further online evaluations. They suggested that in the online evaluation, the participant could act with the robot through the video and microphone, and thus, the participant could get at least a little bit of experience of the interaction: *“If the robot was in front of the video camera, the user could have communicated with it through the microphone. In that case, the situation could have been quite similar to the normal (face to face) testing situation.”* (G3); *“At least the evaluator should interact with the robot in front of the camera, so that the participant could see a person interacting with the robot. That way, s/he could estimate how the interaction would feel like.”* (G3).

## Discussion

The COVID-19 pandemic shifted most of the design projects suddenly into an online mode, placing challenges on Human-Centred Design, which is typically a collaborative and creative activity. Design facilitation and tools such as design canvases are important for creative and collaborative design projects. When designing tangible objects such as physical robots, experiencing their embodiment and presence as part of the design project is important in addition to the presence of co-designers. We found out that designing social robots in an online mode without experiencing them physically was challenging. Based on our findings from short-term and long-term online design projects, we provide a set of practical guidelines for canvas-based facilitation of any kind of collaborative design projects. Furthermore, we propose and discuss the Hybrid Robotics Design Model (HRDM), in which participants are in contact with other people and robots at specific stages of the project, while other stages are conducted fully online by utilizing supporting online tools such as canvases.

### Main Findings about Canvas-Based Teamwork

Based on our findings, canvas-based facilitation seems to have benefits and enablers that can be roughly divided into pragmatic and hedonic, in the same way as Hassenzahl’s user experience model [41] divides the user experiences of a service, concept or product. *Pragmatic aspects* include support for project management, homebase for documentation, and easy perception of the whole process divided into smaller sub-tasks. *Hedonic aspects* include interactional aspects such as social presence, personal canvas identity and expression, feeling of active work and energy, enabling silent communication and emotional layer on communication. Emotional layer is typically naturally present in informal online communications, and a canvas tool may bring it into formal learning settings as well, as the canvas can be made to look playful and inspirational on its appearance.

In our design projects, the experience of the participants appeared to be better in the long-term project, which may be at least partially due to lack of time for online team building in the short-term design workshops. Given the importance of the online team building phase in the Five Stage Model [32–34], even short design workshops should include carefully considered activities for team building and getting familiar with each other. We also observed that the high school workshop participants were overall the most reluctant to communicate via speech or canvas. Based on their feedback, they were the least interested in the topics of the workshops, and moreover, their participation had been arranged via their teacher, whereas the other participant groups were self-registered. This suggests that the challenges that the facilitators encountered with the high school participants were to some degree due to the participants’ lower intrinsic motivation compared to other participant groups.

In their paper, Park and Lim [35] emphasize that online learning environments should support learners’ positive feelings (belonginess, empathy) and decrease negative feelings, such as feelings of isolation, frustration, boredom and anxiety. They proposed a set of design principles to support these goals. Next, we discuss how their design principles are visible on the canvas-based facilitation that we explored. For example, the principles of *positivity* and *playfulness* refer to positive imagination and abilities to play individually and collaboratively while learning. The canvas can be designed to be inspirational, positive and playful workspace as it provides colourful and visual design opportunities and playful elements, such as participants’ avatars, different reactions and rewarding features such as confetti rain. The use of emojis, colours, placement of the elements and personal styles is closely related to the principle of *affinity* [35], which means designing an attractive environment with visually favourable impression. They also relate to the principle of *personalization* [35], which means learners’ freedom to have flexibility in learning inside the environment. Canvas-based facilitation can bring in additional flavour to online collaboration that may otherwise feel unstimulating and boring. Viewing each other working on the canvas can evoke feelings of energy and activity, when collaborators can see cursors and avatars moving, sticky notes being added, and after a short time, the canvas starts looking like a personal and unique workspace due to the placement of the sticky notes, the personal ways people use the canvas, and the colours and materials used on the canvas. Like a real-world physical canvas, the teams can leave their personal and unique “fingerprints” on it. Thus, the principle of *self-disclosure* [35]*,* which refers to the possibilities to feel free about delivering own personal opinion, story or challenges, is encouraged on the canvas. It is important to leave enough freedom for the participants to use the canvas according to their own personal style, and even encourage that, instead of formulating too restricted layouts, flows and ways of interaction. The facilitator can communicate with the team members on canvas by adding sticky notes and some emojis and other visual images, and this style works well as a silent communication, and can be very inspirational for the facilitator as well as the participants. Thus, the canvas provides means for implementing the principle of *humanity* [35], which means delivering a sympathetic instructor formulation with feedback taking into account the human side.

### Practical Guidelines for Canvas-Based Facilitation

In drawing practical guidelines for canvas-based facilitation based on our findings, we utilize the framework by Szumal [6] that divides leadership facilitation into two categories. According to Szumal [6], *interaction facilitation* is a skill of supervisors to utilize people-oriented skills and qualities to encourage supportive, cooperative interactions among their subordinates, thus supporting effective work performance in teams. Interaction facilitation refers to stimulation of group dynamics and communication. *Task facilitation*, on the other hand, is the supervisor’s ability to facilitate the work performance of their subordinates by assisting them in problem-solving and in the implementation of procedural improvements [6]. Task facilitation refers to the management of the goal, task and process itself. Both aspects are important in successful facilitation.

In Table 3, we present a collection of good practices and examples for the facilitators and instructors who want to utilize canvas-based collaboration or collaborative design in their design workshops or courses. According to the two categories of facilitation presented by Szumal [6], we have categorized the guidelines for canvas-based online facilitation under (1) Task related guidelines that relate to how we can facilitate the canvas-based work towards the actual goals of the design project, and (2) Interactional guidelines that concern how we can facilitate the interaction, communication, group dynamics and atmosphere of the project through canvas. The third category presents other good practices and hints that were discovered in our studies.

**Table 3:** Guidelines for practical canvas-based facilitation for any kind of collaborative projects

	Guideline	Example
Task related guidelines I.e. How to structure the canvas and support its use?	Big picture, structure and flow of the project	Structure all the phases/sessions and tasks in one canvas. Keep all phases and tasks in same canvas to help the participants perceive the big picture
	Large goal split into smaller pieces	Split a larger goal into smaller tasks and activities. In a long-term project, make a separate area for each phase on canvas including short tasks
	Support team formation	Formulate a specific task to support initial team formation on canvas
	Empty slots for adding sticky notes	Make empty slot for the participants’ sticky notes next to each task description. Reserve enough space even for creative work such as drawings, images and visuals
	Documentation of the whole process	Encourage the participants for active documentation on canvas. Thus, the canvas will be filled by the participants’ sticky notes, and they can see all the ideas, thoughts and materials in one place
	Supporting materials	Post all related links and materials to the relevant slots
	Schedule and length of each task	Show the schedule of the session on canvas. Show the suggested amount of time that the participants are supposed to spend on each task
	Clear and simple task descriptions	Formulate the task descriptions and instructions as clear and simple as possible
	Wrap-ups and conclusions	Provide a slot for the participants to mark down their conclusions. This helps them to memorize the main points and present their work in the possible common wrap-up of the session
	Learning canvas-based work and culture	Brief the participants about the canvas-based work and its characteristics (why, what, how). Encourage for active use of sticky notes and personal ways of using canvas
	Learning period	Give time for the participants to learn canvas-based working habits and culture. Enable trying out sticky notes and other main features before starting the work
Interactional guidelines I.e. How to boost interaction, collaboration and atmosphere in canvas-based activity?	Visual communication	Use of emoticons and symbols to show reactions, give flavour to your messages and to add an emotional component on your comments
	Textual communication	Use sticky notes actively to activate participants, give feedback, raise questions, provide hints. Keep in mind the importance of praise and rewarding. Place the facilitator’s sticky notes next to the participants’ relevant sticky notes, or where they are moving
	Silent communication	Utilize the benefits of silent communication via sticky notes, especially with well working small teams. There is no need to be present by voice all the time unless the group has problems
	Warm-ups and icebreakers	Create warm-up tasks and ice-breakers (e.g. introductions, creation of ground rules) to get to know each other and to improve atmosphere. As the results are marked on canvas, these can be viewed also afterwards
	Question bank at hand	Post additional questions on the go, have a prepared list of additional questions at hand
	Humour and playfulness	Utilize the canvas’ options for playfulness to create a casual atmosphere: colours, symbols, shapes, rewarding elements (e.g. celebration)
	Canvas presence	Show your presence by commenting actively, moving your cursor on canvas, posting reactions. Active participation on canvas releases the feeling of energy, because we can see everybody moving on canvas, writing sticky notes etc. It gives the feeling of active work and being engaged
	Creative and personal use of canvas leading to unique homebase	Encourage creative and personal use of canvas by using different styles, shapes and colours of sticky notes, organization of the sticky notes, by adding images etc. Leave enough freedom for the participants to use the canvas in their own personal way
	Canvas identity	Use specific colour and shape on sticky notes to express your canvas identity
Other suggestions for canvas-based facilitation E.g. How to improve facilitator’s experience?	Collaborative facilitation	Use several facilitators for the dialogue between facilitators and for improving the facilitator’s experience. Assign different roles to facilitators. Have a dedicated technical facilitator to manage the technical issues. Utilize the dialogue between the facilitators to improve the facilitator’s experience
	Flexible and adaptable facilitation	Every session and set of participants are different. Be prepared to adjust your facilitation style to correspond with the unique needs of each group
	Acceptance of silence	Learn to accept silence and uncomfortable feelings. Give enough time for the participants to think before they respond
	Sharing experiences	Share your experiences with other facilitators after each session to receive feedback and insights
	Combination of tools	Use the suitable combination of tools for facilitation, for example canvas combined with breakout room discussion, Discord or similar for group’s internal communication outside of the facilitated sessions etc

### Hybrid Robotics Design Model (HRDM)

**Fig. 13:**
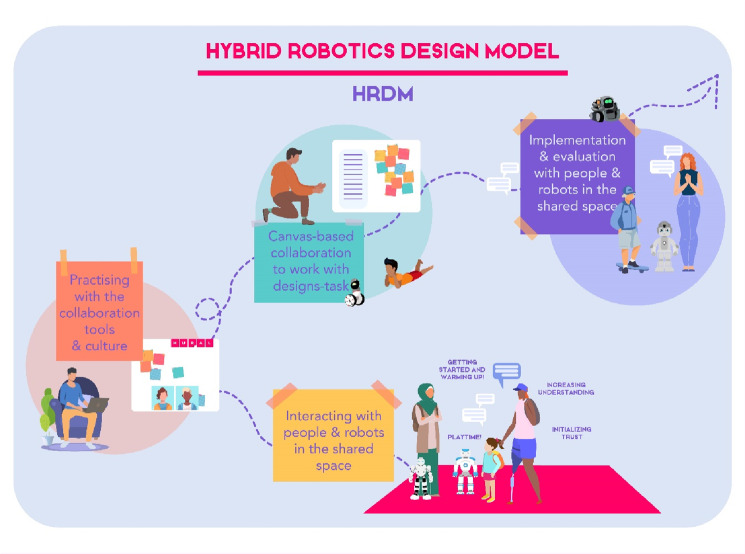
Hybrid Robotics Design Model (HRDM) visualization. Graphic design: Valentina Ramirez Millan

In this section, we discuss design project conduction in a hybrid mode, when it comes to the design of tangible social robots. We suggest *a Hybrid Robotics Design Model (HRDM)* to overcome the barriers of designing tangible robots in a fully online mode. A hybrid mode of conducting design projects may be the dominant model even after pandemic restrictions are lifted due to its benefits for sustainability and inclusiveness of participation. As the physical presence of robots is a special characteristic when interacting and working with tangible robots, it is hard to carry out a fully online robotics design project without missing this fundamental aspect of human–robot interaction. For this reason, it is important to discuss on which stages of the project it is the most necessary to work physically with the robots. It is also important to meet the team members at least at some stages of the project. Figure 13 presents the visualization of the HRDM including the initial phase with the meeting with other people and robots, the middle phases conducted as online work, and the last phases carried out in contact with people and robots again.

#### Meeting People and Robots—Getting Started and Warming-up

Trust is an essential aspect of communication and collaboration, and thus, of effective teamwork [21]. According to Tseng et al. [21], for example the familiarity with the team members, commitment towards the high quality of work, and team cohesion were important factors for building trust with team members. Even though team formation can be facilitated through the canvas via icebreaking activities, meeting the team members physically can build trust between the team members. This need arose specifically with younger workshop participants, with whom it was quite hard to connect with in the online mode. Similarly, Björling and Rose [12] emphasize trust and transparency in participatory design processes especially with teenagers. A similar related finding arose from the longer-term project, as some of the participants revealed their wish of being able to work together physically.

In our studies, we learned that participants’ understanding of what robots are and what they can do remained too abstract and shallow without experiencing the interaction with robots in the physical world. This observation concerned especially the short-term projects. Thus, we suggest that in the beginning of any robotic design workshop or project, a meeting with the robots and other participants is organized. This suggestion is in line with Salmon’s Access and Motivation phase [32–34], where the purpose of the first phases is to set safe and good grounds for learning and collaborative work. At this phase, the participants also need to get familiar with their canvas, which will act as a home base during the whole project. The canvas-based culture and communication need to be introduced to the participants at this phase, as well as the other tools in use.

#### Canvas-Based Collaboration—Planning and Ideation

At this phase, the work can continue online on canvas and by utilizing other suitable online tools. The canvas needs to be designed in a way that supports each phase and task of the workshop or project. Testing the usability and user experience of the canvas design before giving participants access to it is highly recommended. The task descriptions need to be self-explanatory and clear, and there should be enough space for the sticky notes and other materials. As participants document their thinking and ideas on the canvas, all phases of the project get documented in the same place and the participants and the facilitator can view any phase as needed. A well-structured canvas supports planning and ideation. The additional benefits of the canvas are the inspirational nature of the canvas, the presence of other people which can be felt through the canvas, and canvas-based communication, which can take place silently and unobtrusively between the participants and the facilitator. The canvas-based communication can be inspirational and dynamic and boost the collaborative and active atmosphere.

#### Interaction with People and Robots—Prototyping and Evaluation

After the planning and concept design work is done, the design team would benefit from meeting the physical robot again. In our longer-term design project, we learned that some students expressed how interesting it was to make the implementation of the concept on the actual robot rather than as a scenario or storyboarding technique. It is challenging or even impossible to demonstrate the human–robot interaction with the physical robot in online settings, and by conducting this phase on fully online settings we easily miss a fundamental aspect of the design project. The physical presence is an essential factor in the human–robot interaction. The same challenge seemed to apply to the user evaluation phase, where it was hard to evaluate the human–robot interaction in online settings. Thus, we recommend integrating contact work with the robot(s) in the prototyping/implementation phase, and with the robot(s) and user study participants in the evaluation part.

#### Discussion on the HRDM

The Hybrid Robotics Design Model is an initial model of how we could ideally combine contact sessions with people and robots to online work phases, which would take place for example by utilizing canvas-based collaboration and other suitable online tools. The model was developed due to the observed challenges when designing tangible social robots in fully online settings. As this model development is in its early stages, we suggest that different stakeholders can take inspiration out of it, and adjust the model based on their own needs and experiences as fit. We hope that the model and its suitability and adjustability for the robotic design projects will be discussed in the future work, as we and other robotic designers will get more experience in the robotic design projects conducted in the hybrid mode. Different optimal combinations of contact and online phases may suit for different conditions and goals. As future work, we aim at developing the HRDM further, and apply it in practice in several different kinds of robotic projects.

### Limitations and Future Work

For this work we were able to collect participant feedback only via survey forms, while facilitators’ experiences were gathered as reflective writings and transcribed discussions. While the surveys allowed for open-ended responses, the data on the participants’ experiences remains less elaborate and subject to researchers’ interpretations. In this sense, the participants’ perspective on canvas-based collaborative design work is limited to a degree.

To better address user needs during collaborative canvas-based practices, it would be interesting to explore in further detail what are the opportunities and barriers in online canvases for individual participation. To improve the design of collaborative online tools, one approach could be to study how people utilize the digital canvas to communicate and collaborate online, revealing how different features are used or dismissed in different situations. In future work, a study comparing online and in-person design activities on social robotics would be beneficial in order to get comparable knowledge about these two modes.

## Conclusions

Most design activities and design projects had to be switched to an online mode almost overnight when the COVID-19 pandemic began. Online robotics design projects are challenging due to the embodied existence of tangible robots. Even though the world seems to be recovering from the pandemic and work is returning to offices and classrooms, there are good learnings from the work carried out online, which can be continued to some extent. Hybrid ways of working can be utilized even in the design projects of tangible robots, entailing physical meetings at the meaningful phases of the project and otherwise collaborating online by utilizing a set of suitable tools.

In this article we have presented examples of design activities that utilized online canvases for facilitation and learning. We collected mainly qualitative data including written reflections of the participants and facilitators. We have proposed practical guidelines for canvas-based facilitation to support creative and collaborative design projects. The guidelines have been presented in three categories: task-related guidelines, interactional guidelines, and other guidelines. We have also suggested and discussed a Hybrid Robotic Design Model (HRDM), which can be utilized and adapted to several types of robotic design projects. Hybrid mode of work enables higher accessibility, equality and inclusiveness for participants of design projects compared to work that takes place solely in a physical environment. Still, conducting the critical phases of the project in a physical environment with tangible robots enables better understanding of the fundamental aspects of human–robot interaction. We will utilize the HRDM and canvas-based facilitation in our upcoming design workshops and courses, and we encourage other researchers and designers to adapt them to their own design projects.

## Data Availability

The datasets generated during the current study are available from the corresponding author on reasonable request.
